# Supplementary material for: Structural basis of RNA polymerase II transcription on the chromatosome containing linker histone H1
Source: Nat Commun. 2022 Nov 26;13:7287. doi: 10.1038/s41467-022-35003-z (PMC9701232; doi:10.1038/s41467-022-35003-z)
Supplement: Supplementary file 1 — Supplementary information [file 41467_2022_35003_MOESM1_ESM.pdf]

Structural basis of RNA polymerase II transcription on the chromatosome containing linker histone H1

Rina Hirano[1,3], Haruhiko Ehara[2,3], Tomoya Kujirai[1,2], Tamami Uejima[2], Yoshimasa Takizawa[1], Shun-ichi Sekine[2]*, Hitoshi Kurumizaka[1,2,]*

[1] Laboratory of Chromatin Structure and Function, Institute for Quantitative Biosciences, The University of Tokyo, 1-1-1 Yayoi, Bunkyo-ku, Tokyo 113-0032, Japan.
[2] RIKEN Center for Biosystems Dynamics Research, 1-7-22 Suehiro-cho, Tsurumi-ku, Yokohama 230-0045, Japan.

Supplementary Table 1
Cryo-EM data collection, refinement, and validation statistics for RNAPII-chromatosome complexes

	form I (EMDB-34415) (PDB 8H0V)	form II (EMDB-34416) (PDB 8H0W)
**Data collection and processing**		
Magnification	81,000	
Voltage (kV)	300	
Electron exposure ($e^{-}/Å^2$)	56.2	
Defocus range (μm)	1.2 - 2.3	
Pixel size (Å)	1.07	
Symmetry imposed	C1	C1
Initial particle images (no.)	402427	402427
Final particle images (no.)	75631	16916
Map resolution (Å) (FSC=0.143)		
Overall	3.8	4.6
RNAP	3.3	4.2
Nucleosome	3.5	4.5
Map resolution range (Å)	3.1-26.8	3.4-27.0
**Refinement**		
Initial model used (PDB code)	5XOG, 3LZ0, 4QLC	
Map sharpening B factor ($Å^2$)	-86.7	-66.0
Model composition		
Non-hydrogen atoms	46571	46364
Protein residues	4766	4766
Ligands	Zn:8 Mg:1	Zn:8 Mg:1
B factors ($Å^2$)		
Protein	291.5	289.9
Ligand	308.4	329.1
R.m.s. deviations		
Bond lengths (Å)	0.006	0.006
Bond angles (°)	0.872	0.819
Validation		
MolProbity score	1.78	1.77
Clashscore	9.96	9.58
Rotamer outliers (%)	0.05	0.02
Ramachandran plot		
Favored (%)	96.13	96.09
Allowed (%)	3.79	3.83
Disallowed (%)	0.09	0.09

Supplementary Figures

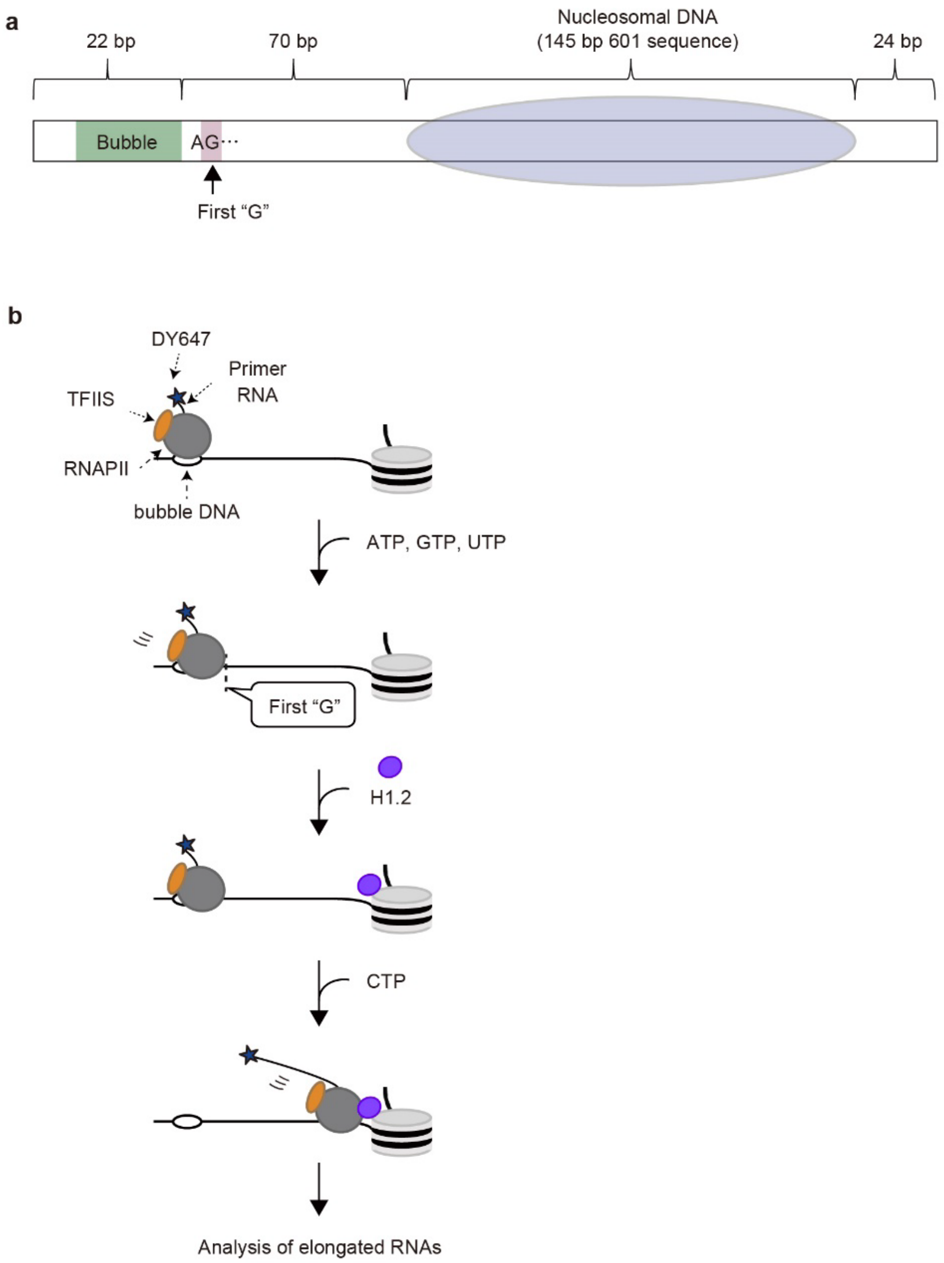

Supplementary Figure 1
Schematic representation of the transcription assay. (a) Graphical representation of the template for chromatosome transcription by RNAPII. (b) Schematic representation of the transcription assay with the chromatosome. RNAPII was loaded on the bubble DNA region together with the DY647-labeled primer RNA, in the presence of the transcription elongation factor TFIIS. The transcription reaction was initiated by adding ATP, GTP, and UTP. The RNAPII stalled at the "first G" position, because of the absence of its complementary nucleotide, CTP. This step suppressed the RNAPII and primer RNA dissociation from the template nucleosome. Histone H1.2 was then added

to the reaction mixture, and the chromatosome was assembled by the H1-nucleosome binding. The transcription reaction was re-initiated by adding CTP, and the resulting RNAPII-chromatosome complexes were analyzed.

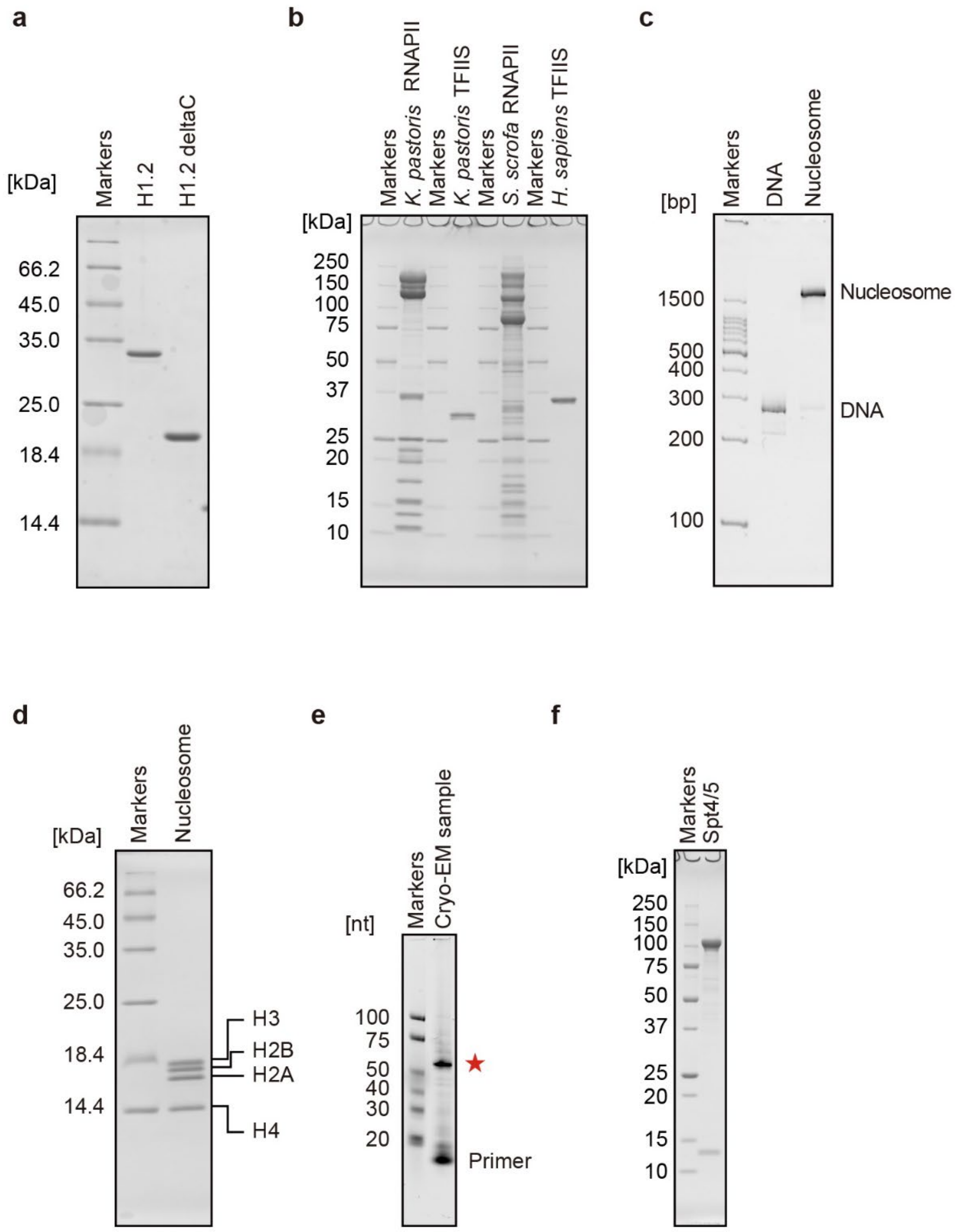

Supplementary Figure 2
Sample preparation. (a) Purified H1.2 and H1.2 deltaC were analyzed by 16% SDS-PAGE with Coomassie Brilliant Blue (CBB) staining. (b) Purified *K. pastoris* RNAPII, *S. scrofa* RNAPII, *K. pastoris* TFIIS, and *H. sapiens* TFIIS were analyzed by 10-20% SDS-PAGE with CBB staining. (c) The template nucleosome was reconstituted,

purified, and analyzed by native polyacrylamide gel electrophoresis with ethidium bromide staining. (d) The histone composition was analyzed by SDS 18% polyacrylamide gel electrophoresis with CBB staining. (e) The RNA products of the transcribing RNAPII-chromatosome complex, prepared by the GraFix method for cryo-EM analysis, were analyzed by denaturing gel electrophoresis. (f) Purified *K. pastoris* Spt4/5 was analyzed by 10-20% SDS-PAGE with CBB staining.

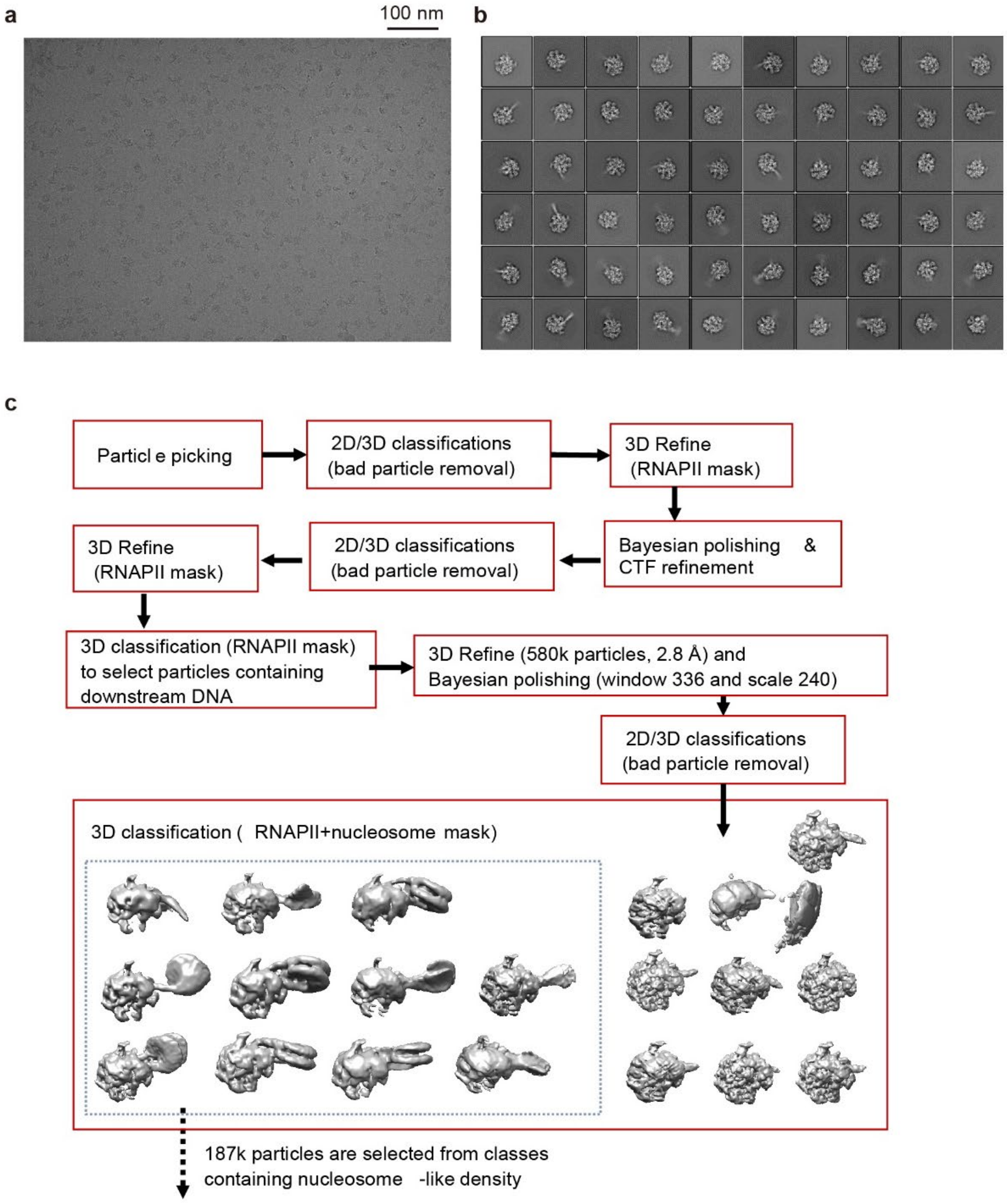
a
100 nm
b
c
Particl e picking
2D/3D classifications
(bad particle removal)
3D Refine
(RNAPII mask)
Bayesian polishing &
CTF refinement
2D/3D classifications
(bad particle removal)
3D Refine
(RNAPII mask)
3D classification (RNAPII mask)
to select particles containing
downstream DNA
3D Refine (580k particles, 2.8 Å) and
Bayesian polishing (window 336 and scale 240)
2D/3D classifications
(bad particle removal)
3D classification (RNAPII+nucleosome mask)
187k particles are selected from classes
containing nucleosome -like density

Supplementary Figure 3 (continued)

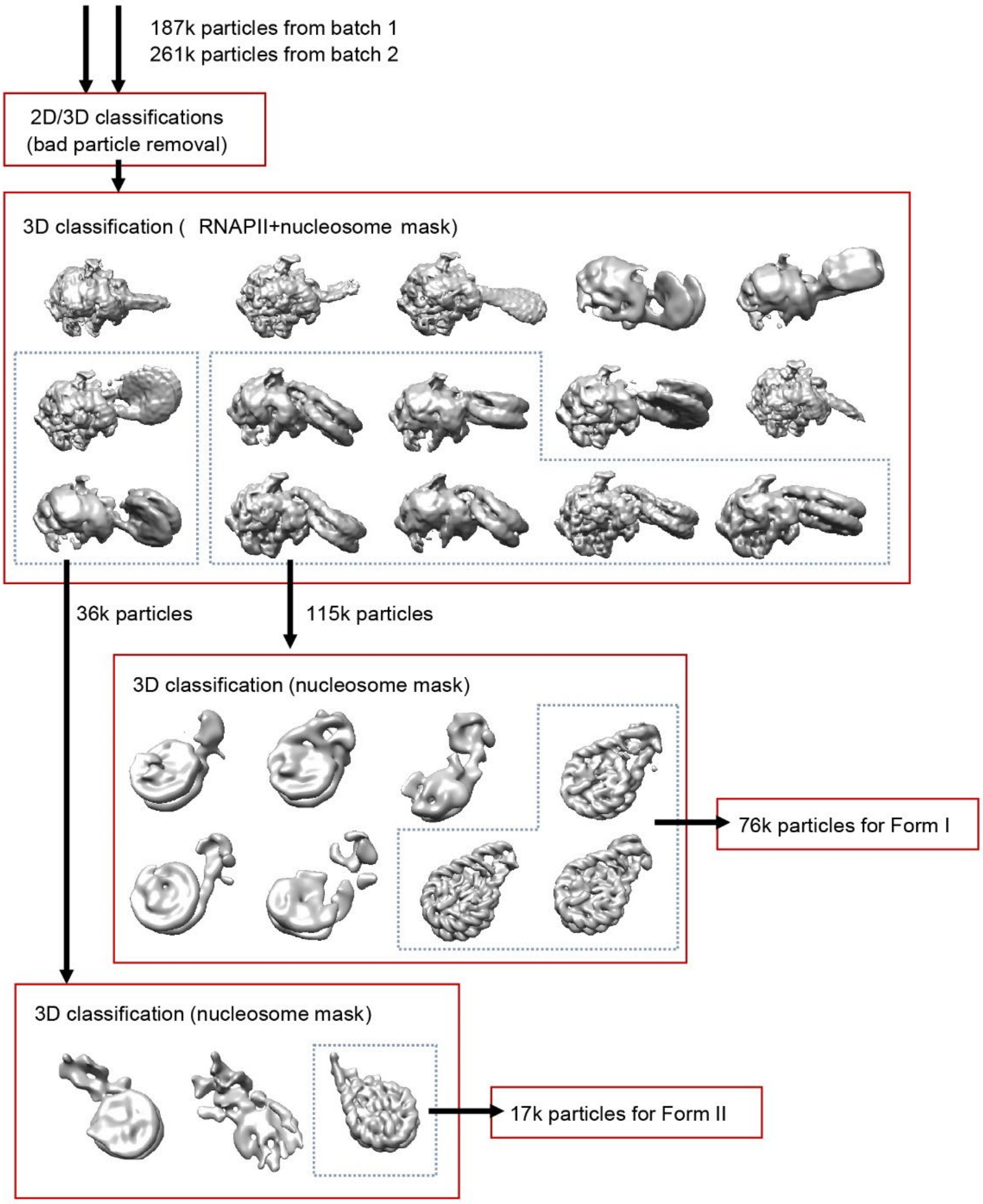

Supplementary Figure 3
Cryo-EM data collection and initial image processing. (a) A representative image from the 8051 cryo-EM micrographs in the dataset. (b) Representative 2D class averages from the reference-free 2D classification, calculated from particles containing strong downstream DNA density. (c) Flowchart of the initial stage of the image processing (data processing batch 1).

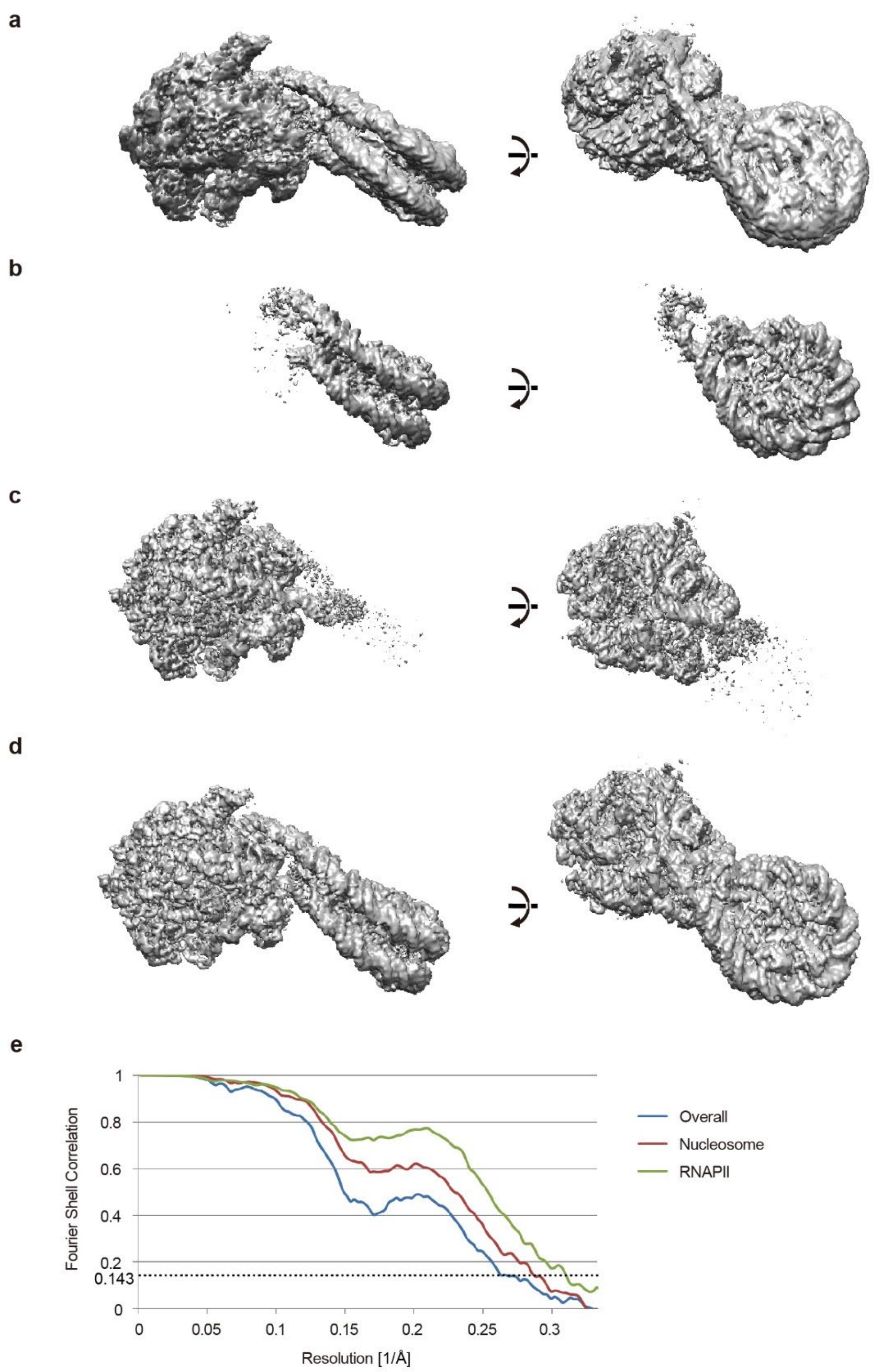

Supplementary Figure 4
Cryo-EM reconstruction of the form I complex. Cryo-EM maps of (a) the overall reconstruction, (b) the nucleosome reconstruction, and (c) the RNAPII reconstruction. (d) The composite map was calculated from the three reconstructions. (e) Fourier shell correlation curves for the cryo-EM reconstructions.

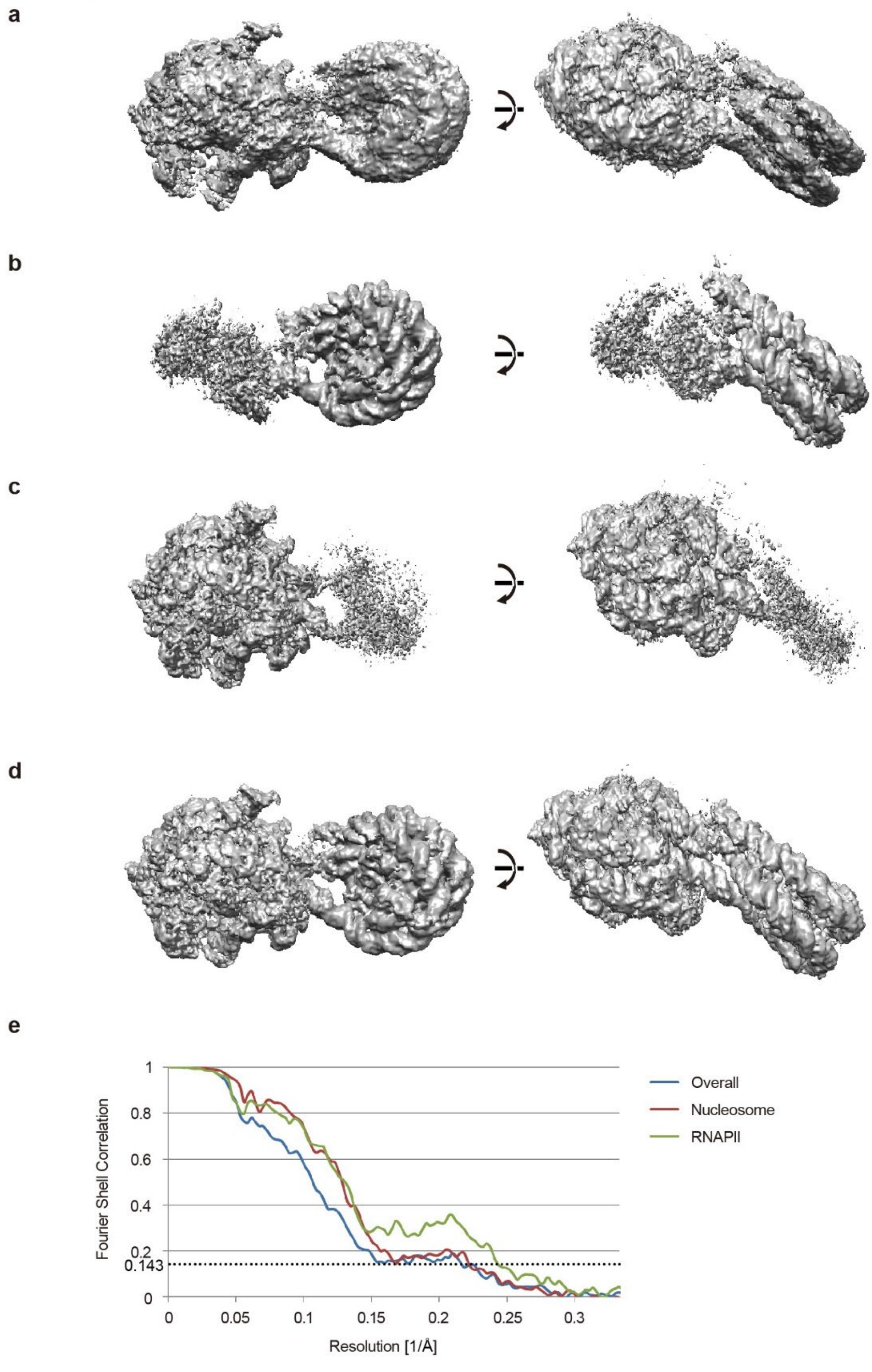

Supplementary Figure 5
Cryo-EM reconstruction of the form II complex. The cryo-EM maps of (a) the overall reconstruction, (b) the nucleosome reconstruction, and (c) the RNAPII reconstruction. (d) The composite map was calculated from the three reconstructions. (e) Fourier shell correlation curves for the cryo-EM reconstructions.

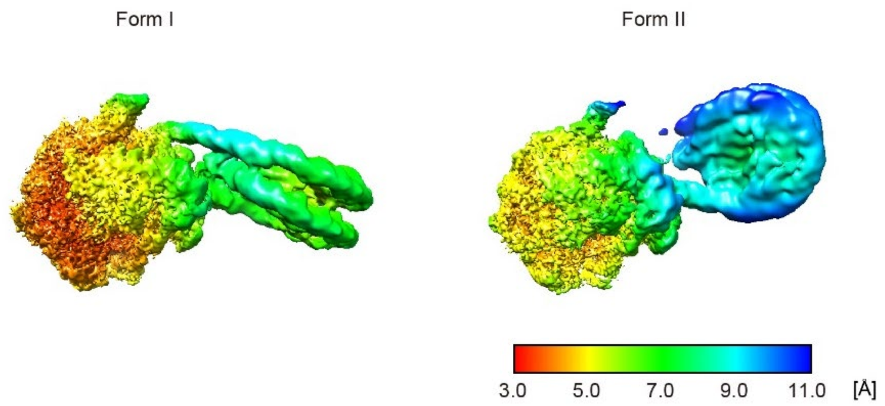

Supplementary Figure 6
Local resolution maps of overall reconstructions. Local resolution values were calculated with 35 Å sampling, and plotted on locally-filtered maps calculated by relion.postprocess.

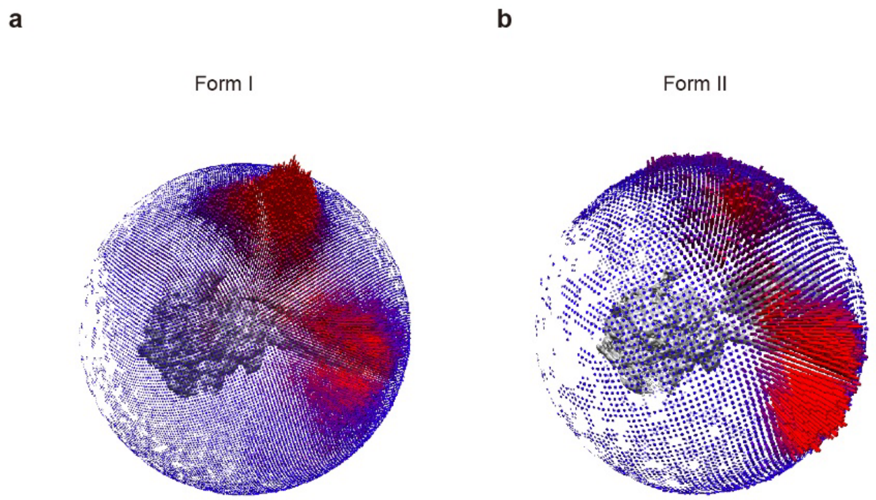

Supplementary Figure 7
Euler distribution plots of overall reconstructions.

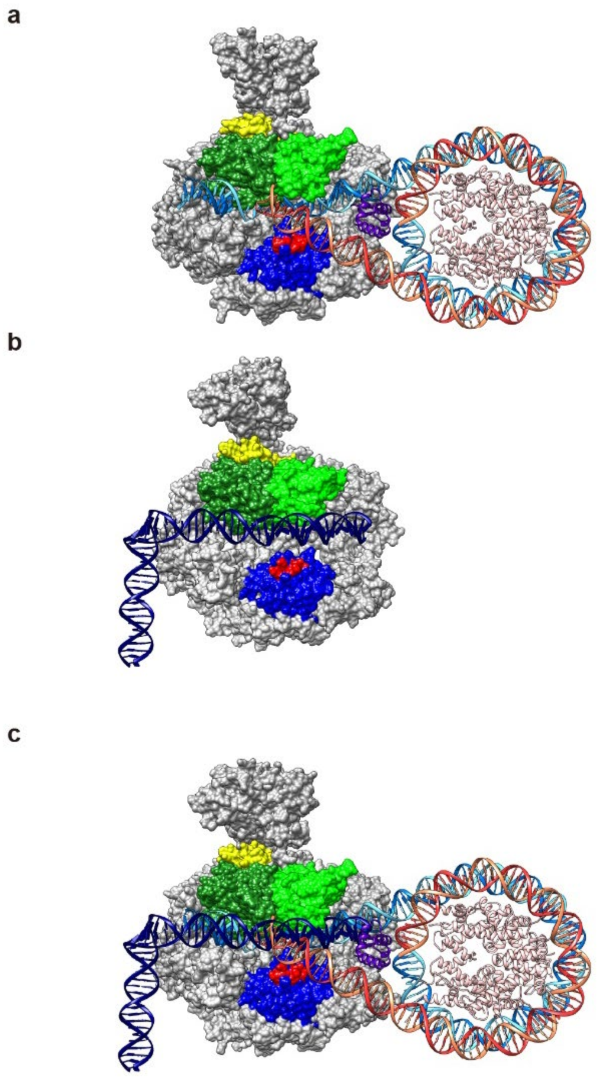

Supplementary Figure 8
The exit linker DNA in the RNAPII-chromatosome complex (form I) contacts the RNAPII surface in a similar manner to the RNAPII DNA-binding cleft in the preinitiation complex. (a) Cryo-EM structure of the RNAPII-chromatosome complex (form I). Color codes are the same as those in Fig. 2. (b) Cryo-EM structure of RNAPII and DNA in the preinitiation complex (PDB ID: 5FZ5). The DNA is represented as a dark blue ribbon. (c) Superimposition of the form I complex and the RNAPII preinitiation complex.

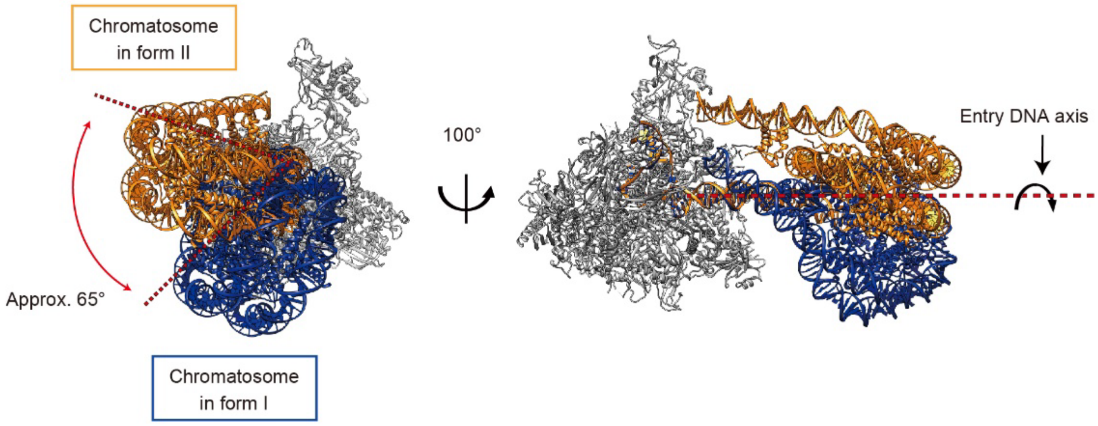

Supplementary Figure 9
Orientations of the chromatosome relative to RNAPII in the form I and form II complexes. The RNAPII models in the form I and form II complexes were fitted to compare the relative locations of the chromatosomes. The chromatosomes in the form I and form II complexes are colored blue and orange, respectively. The chromatosome arrangement of form I and form II relative to the RNAPII is rotated around the entry DNA axis by approximately 65°. The entry DNA axis is indicated by the dashed line.

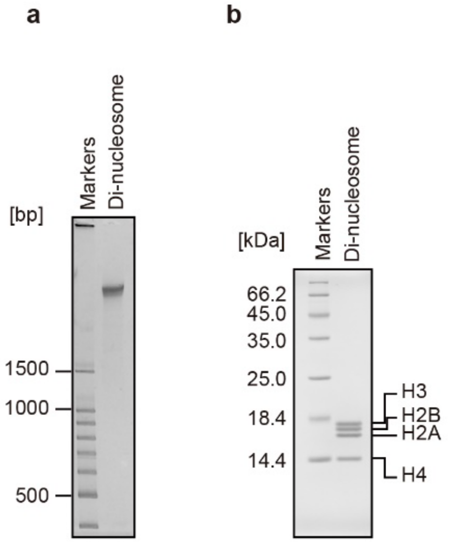

Supplementary Figure 10
Preparation of the di-nucleosome template. (a) The di-nucleosome template was reconstituted, purified, and analyzed by native polyacrylamide gel electrophoresis with ethidium bromide staining. (b) The histone composition was analyzed by SDS 18% polyacrylamide gel electrophoresis with CBB staining.

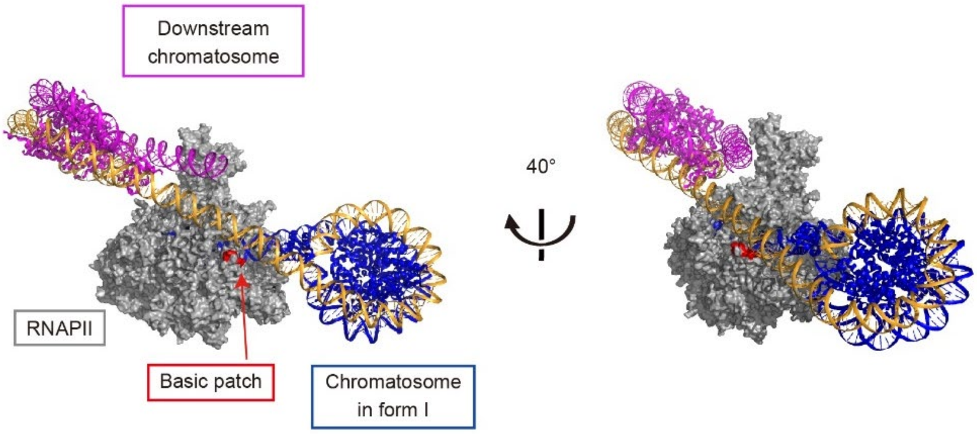

Supplementary Figure 11
Model of the di-chromatosome complexed with RNAPII. The downstream chromatosome (PDB ID: 7K5Y) was placed in the form I complex. The model contains a 48 bp linker DNA between the two nucleosomes. The DNA segment from the dyad of the upstream chromatosome to the dyad of the downstream chromatosome is colored orange.

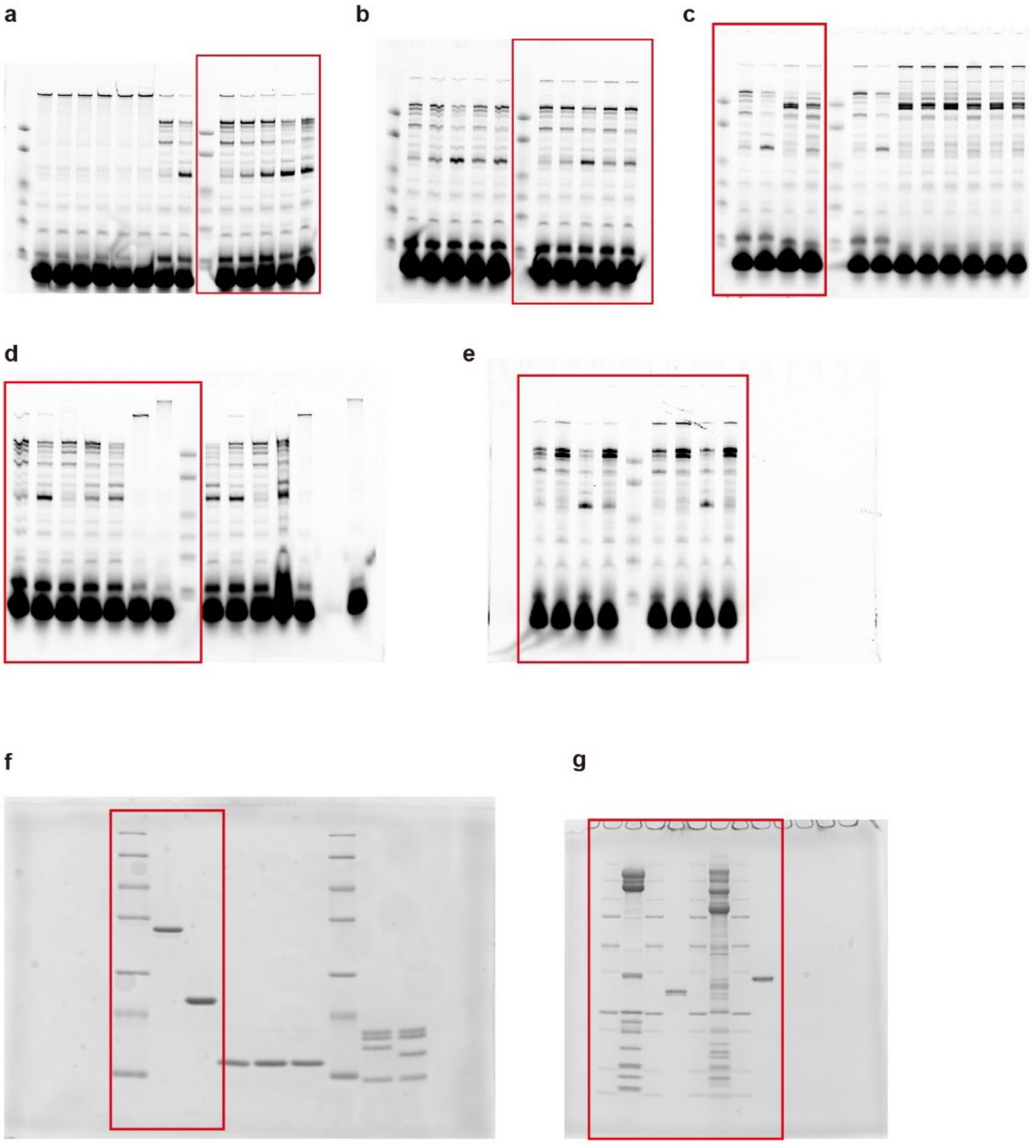
a
b
c
d
e
f
g

Supplementary Figure 12 (continued)

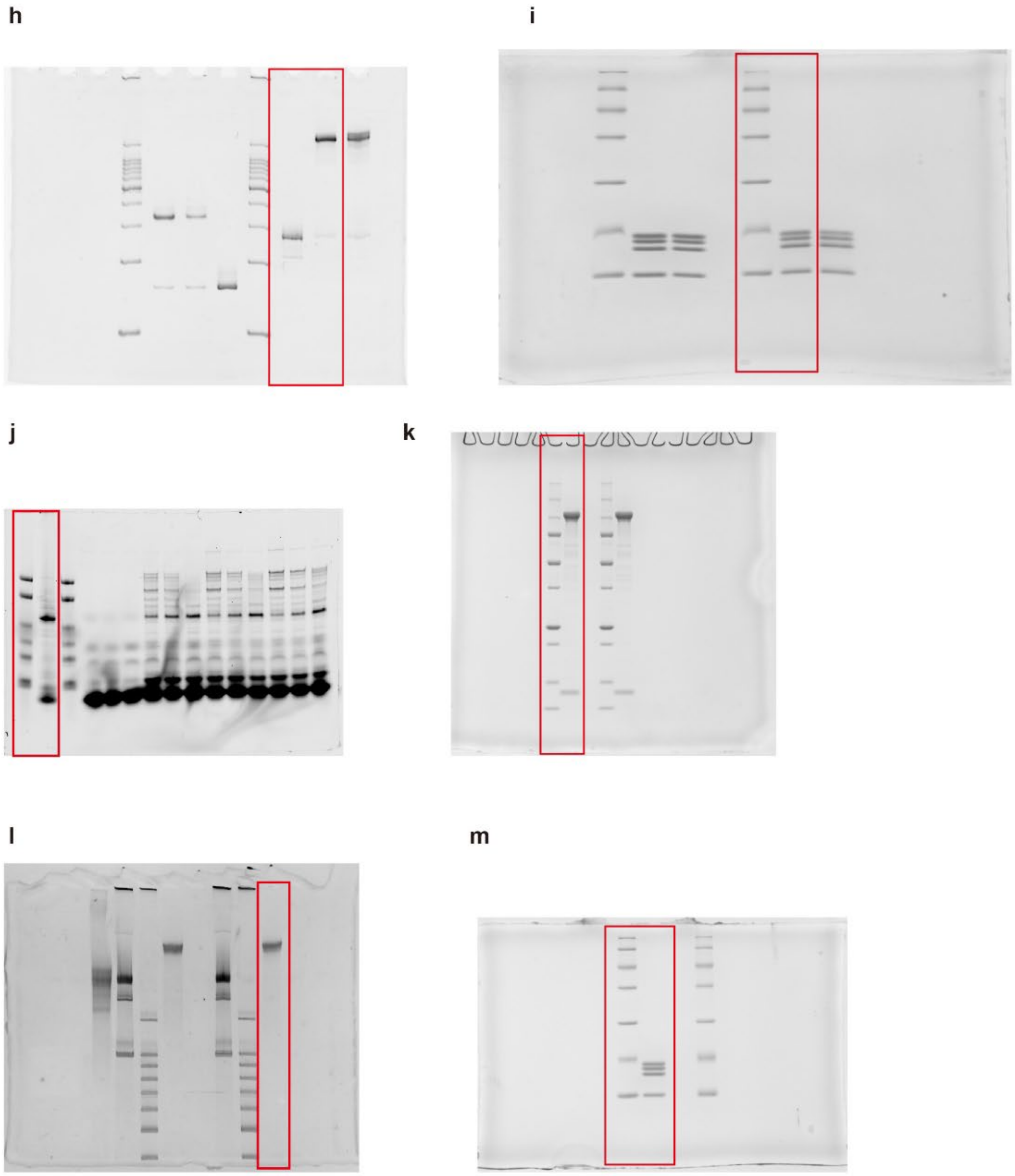

Supplementary Figure 12. Full images.
The full images of (a) Fig. 1c, (b) Fig. 1d, (c) Fig. 1e, (d) Fig. 4b, (e) Fig. 5b, (f) Supplementary Fig. 2a, (g) Supplementary Fig. 2b, (h) Supplementary Fig. 2c, (i) Supplementary Fig. 2d, (j) Supplementary Fig. 2e, (k) Supplementary Fig. 2f, (l) Supplementary Fig. 10a, and (m) Supplementary Fig. 10b.
