# Supplementary material for: Structural basis of RNA polymerase II transcription on the chromatosome containing linker histone H1
Source: Nat Commun. 2022 Nov 26;13:7287. doi: 10.1038/s41467-022-35003-z (PMC9701232; doi:10.1038/s41467-022-35003-z)
Supplement: Supplementary file 3 — Reporting Summary [file 41467_2022_35003_MOESM3_ESM.pdf]

Corresponding author(s): Shun-ichi Sekine, Hitoshi Kurumizaka

Last updated by author(s): Nov 14, 2022

Reporting Summary

Nature Portfolio wishes to improve the reproducibility of the work that we publish. This form provides structure for consistency and transparency in reporting. For further information on Nature Portfolio policies, see our Editorial Policies and the Editorial Policy Checklist.

Statistics

For all statistical analyses, confirm that the following items are present in the figure legend, table legend, main text, or Methods section.

n/a	Confirmed	
☒	☐	The exact sample size (*n*) for each experimental group/condition, given as a discrete number and unit of measurement
☐	☒	A statement on whether measurements were taken from distinct samples or whether the same sample was measured repeatedly
☒	☐	The statistical test(s) used AND whether they are one- or two-sided<br>*Only common tests should be described solely by name; describe more complex techniques in the Methods section.*
☒	☐	A description of all covariates tested
☒	☐	A description of any assumptions or corrections, such as tests of normality and adjustment for multiple comparisons
☒	☐	A full description of the statistical parameters including central tendency (e.g. means) or other basic estimates (e.g. regression coefficient) AND variation (e.g. standard deviation) or associated estimates of uncertainty (e.g. confidence intervals)
☒	☐	For null hypothesis testing, the test statistic (e.g. *F*, *t*, *r*) with confidence intervals, effect sizes, degrees of freedom and *P* value noted<br>*Give P values as exact values whenever suitable.*
☒	☐	For Bayesian analysis, information on the choice of priors and Markov chain Monte Carlo settings
☒	☐	For hierarchical and complex designs, identification of the appropriate level for tests and full reporting of outcomes
☒	☐	Estimates of effect sizes (e.g. Cohen's *d*, Pearson's *r*), indicating how they were calculated

Our web collection on statistics for biologists contains articles on many of the points above.

Software and code

Policy information about availability of computer code

Data collection	SerialEM ver.3
Data analysis	Relion 3.1, Gautomatch 0.56, Gctf 1.06, topaz 0.2.5, Wincoot 0.9.6, ChimeraX 1.2.5, ISOLDE 1.2.0, Chimera 1.16, PyMOL 2.5.2, PHENIX 1.19.2

For manuscripts utilizing custom algorithms or software that are central to the research but not yet described in published literature, software must be made available to editors and reviewers. We strongly encourage code deposition in a community repository (e.g. GitHub). See the Nature Portfolio guidelines for submitting code & software for further information.

Data

Policy information about availability of data

All manuscripts must include a data availability statement. This statement should provide the following information, where applicable:

- Accession codes, unique identifiers, or web links for publicly available datasets
- A description of any restrictions on data availability
- For clinical datasets or third party data, please ensure that the statement adheres to our policy

The cryo-EM reconstructions and atomic models of the RNA polymerase II-nucleosome-H1 complexes generated in this study have been deposited in the Electron Microscopy Data Bank and the Protein Data Bank, under the accession codes (EMD-34415 [https://www.ebi.ac.uk/emdb/EMD-34415] and PDB ID 8H0V [https://www.rcsb.org/structure/unreleased/8H0V] for the RNAPII-nucleosome-H1 form I complex, and EMD-34416 [https://www.ebi.ac.uk/emdb/EMD-34416] and PDB ID 8H0W [https://www.rcsb.org/structure/unreleased/8H0W] for the RNAPII-nucleosome H1 form II complex. The structures used in this study can be found in the Protein Data Bank under the accession codes: 5XOG [https://www.rcsb.org/structure/5XOG], 3LZ0 [https://www.rcsb.org/structure/3LZ0], 4QLC [https://

www.rcsb.org/structure/4QLC], 7K5Y [https://www.rcsb.org/structure/7K5Y].

Field-specific reporting

Please select the one below that is the best fit for your research. If you are not sure, read the appropriate sections before making your selection.

- [x] Life sciences
- [ ] Behavioural & social sciences
- [ ] Ecological, evolutionary & environmental sciences

For a reference copy of the document with all sections, see nature.com/documents/nr-reporting-summary-flat.pdf

Life sciences study design

All studies must disclose on these points even when the disclosure is negative.

Sample size	Since our study does not contain statistical tests, we did not perform sample-size calculation. For all transcription assays, we peformed at least three independent experiments, which would be sufficient to check the reproducibility. For cryo-EM analysis, we used 8051 micrographs for structure reconstitution, which was sufficient for structure reconstruction at the resolution required for the discussion in this paper.
Data exclusions	No data were excluded from the analyses.
Replication	The reproducibility for all transcription assays was confirmed by more than three independent experiments. The cryo-EM data set was collected from one grid.
Randomization	Randomization was not performed. Randomization is not necessary for the discussion in this paper.
Blinding	Biochemical and cryo-EM analyses are not blinded. Researchers' biases will not occur in our biochemical assays and cryo-EM structural analyses.

Reporting for specific materials, systems and methods

We require information from authors about some types of materials, experimental systems and methods used in many studies. Here, indicate whether each material, system or method listed is relevant to your study. If you are not sure if a list item applies to your research, read the appropriate section before selecting a response.

Materials & experimental systems

n/a	Involved in the study	
[x]	[ ]	Antibodies
[x]	[ ]	Eukaryotic cell lines
[x]	[ ]	Palaeontology and archaeology
[x]	[ ]	Animals and other organisms
[x]	[ ]	Human research participants
[x]	[ ]	Clinical data
[x]	[ ]	Dual use research of concern

Methods

n/a	Involved in the study	
[x]	[ ]	ChIP-seq
[x]	[ ]	Flow cytometry
[x]	[ ]	MRI-based neuroimaging
